# Modelling peeling- and pressure-driven propagation of arterial dissection

**DOI:** 10.1007/s10665-017-9948-0

**Published:** 2017-12-13

**Authors:** Lei Wang, Nicholas A. Hill, Steven M. Roper, Xiaoyu Luo

**Affiliations:** 1 School of Mathematics and Statistics, University of Glasgow, University Place, Glasgow, G12 8QQ UK; 2 Department of Engineering, Durham University, Durham, DH1 3LE UK

**Keywords:** Arterial dissection, Cohesive traction-separation law, Collagen fibres, HGO constitutive law, Tear, Wall buckling, XFEM

## Abstract

An arterial dissection is a longitudinal tear in the vessel wall, which can create a false lumen for blood flow and may propagate quickly, leading to death. We employ a computational model for a dissection using the extended finite element method with a cohesive traction-separation law for the tear faces. The arterial wall is described by the anisotropic hyperelastic Holzapfel–Gasser–Ogden material model that accounts for collagen fibres and ground matrix, while the evolution of damage is governed by a linear cohesive traction-separation law. We simulate propagation in both peeling and pressure-loading tests. For peeling tests, we consider strips and discs cut from the arterial wall. Propagation is found to occur preferentially along the material axes with the greatest stiffness, which are determined by the fibre orientation. In the case of pressure-driven propagation, we examine a cylindrical model, with an initial tear in the shape of an arc. Long and shallow dissections lead to buckling of the inner wall between the true lumen and the dissection. The various buckling configurations closely match those seen in clinical CT scans. Our results also indicate that a deeper tear is more likely to propagate.

## Introduction

Arterial dissection is a cardiovascular disease with a high mortality rate. An arterial dissection begins when a defect arises in the intimal layer of the arterial wall. The defect tends to grow to become a tear in the medial layer; blood leaks into the tear to create a *false lumen*. Subject to loading, the tear may propagate along the media, which can lead to death as a result of decreased blood supply to other major organs, damage to the aortic valve, and sometimes rupture of the artery [1, 2]. In this paper, we develop computational models of arterial dissection to understand the mechanical issues surrounding the development of an existing tear in the arterial wall. We define two types of propagation of a dissection. We do not consider the dynamics of tear propagation; instead, we are concerned with identifying a critical value of the loading parameter at which tear propagation might be expected to occur. The first, which we identify as ‘peeling-driven’ propagation, has displacement control on the boundaries of sections of the arterial wall and has zero traction on the tear faces. The second, which we identify as ‘pressure-driven’ propagation, has pressure loading on the tear faces and the lumen of the blood vessel.

Peeling-driven propagation describes a standard test for the characterisation of material strength, in which a torn sample is subjected to displacement boundary conditions. Gasser and Holzapfel [3] developed an eXtended Finite Element Method (XFEM) model for such a configuration. Ferrara and Pandolfi [4] used the conventional finite element method to model the same problem. Both groups used the Holzapfel–Gasser–Ogden (HGO) strain-energy function to describe the mechanical response of the anisotropic arterial wall, but different cohesive traction-separation laws were used for the evolution of the tear; it is an exponential isotropic function of separation in [3], but a linear function with fibre-dependent directional preference in [4]. These computational models were used to model peeling experiments on the human aortic media by Sommer et al. [5]. Similar work was carried out for peeling experiments on human coronary [6] and carotid [7] arteries.

Pressure-driven propagation is an alternative experimental protocol to study the mechanics underlying arterial dissection. Carson et al. [8] measured the value of pressure to initiate a dissection in strips of porcine upper thoracic aortas, as well as curves of pressure against the volume of the false lumen. Taking a slab of tissue from the longitudinal direction of the aortas with three layers (intima, media and adventitia), and infusing a fluid at a constant flow rate into the media, they found that as the pressure increased to 77 kPa, a bleb formed, and the tear started to propagate, at which point the flow into the dissection was halted. Propagation continued, with the pressure decreasing as the volume increased before stopping at a pressure of around 25 kPa. Tam et al. [9] performed a pressure-driven tear propagation in the porcine aortic wall assuming the artery natural geometry to be a cylindrical tube. An initial false lumen was created by injecting saline solution into the media, a radial–circumferential slit was introduced to connect the true and false lumens, which ensures the same pressure in both lumens. Each aorta was then submerged in 0.9% saline and pressurised under static conditions. The pressure was increased up to the critical value corresponding to the onset of propagation. Introducing the initial tear at different radial distances (depths) from the inner radius of the aorta, they found that the critical pressure decreased almost linearly with depth.

Pressure-driven propagation is closer to *in vivo* loading environment of the arterial dissection, as well as to hypertension-induced dissection *in vivo* [10], than the peeling-driven case. However, there are few modelling studies on pressure-driven dissection propagation. Rajagopal et al. [11] presented a mathematical model describing mechanical factors in the initiation and propagation of a dissection. They postulated that the haemodynamic conditions that render the aorta susceptible to the initiation of dissection are elevated maximum systolic and mean aortic blood pressure, whereas the haemodynamic conditions that facilitate propagation of a dissection are elevated pulse pressure and heart rate. Although the equations for the nonlinear viscoelasticity of the arterial wall and for delamination of the individual layers composing the aortic wall were also included, their model has not been applied to any examples. In [12], we computed the energy release rate for a mathematical model of pressure-driven dissection propagation in two-dimensional arterial wall strips, and observed that the connective tissue may result in dissection arrest, i.e. the tear propagation can be stopped after first spread due to the support of the connective tissues. In other words, diseased connective tissues enhances the risk of dissection. Recently, we investigated the effect of residual stress on the critical pressure for dissection propagation in [13], which demonstrated that residual stress can elevate the critical pressure and thus lower the risk propagation. Gültekin et al. [14] used a numerical phase-field approach and, after parameter fitting, found good agreement with experiments by Sommer et al. [15] on shear-loading tests of both aneurysmatic and dissected human thoracic aortas that showed that the orthotropic media has significantly greater resistance to out-of-plane than to in-plane shear loading. Sommer et al. also found that aortas with dissections had on average much less mechanical strength than aneurysmal specimens. Tsamis et al. [16] conducted a major review of published data and concluded that high blood pressure and degradation of the both elastin and collagen in the wall contribute to the formation of dissections in the thoracic aorta.

In this paper, we simulate dissection propagation subject to both peeling and pressure. We assume the arterial wall to be an fibre-reinforced hyperelastic incompressible material, and employ the HGO strain-energy function. Tear initiation and propagation are governed by a linear cohesive traction-separation law. Solutions are computed using the XFEM [17] implemented in Abaqus [18]. For illustration, the simulations of peeling-driven tear propagation in strips and discs of arterial wall samples are used to study the direction of tear propagation. Both plane-strain and three-dimensional models are developed. The conclusions from running these models suggest that the tear tends to propagate along the direction of fibre orientation. Simulations of pressure-driven tear propagation in the cross-section of two-layer arterial wall are performed to investigate the effects of tear length and depth on the critical condition and propagation. The results show that the collapse of wall flap (the layer between the true and false lumina) for a long and shallow dissection may slow down the tear propagation. These deformed buckling configurations are not uncommon, as demonstrated by a number of published *in vivo* medical images. We conclude that flap thickness may be an important factor in predicting the evolution of dissections.

The paper is organised as follows. In Sect. 2, the mathematical models are described. Section 3 gives the results of the peeling-driven case, and Sect. 4 studies pressure-driven propagation. The conclusions are drawn in the final section.

## The computational model

### Material

We assume the arterial wall to be a hyperelastic incompressible material that can be described by the HGO strain-energy function [19]:

1 $$\begin{aligned} \varPsi \equiv \varPsi _{m}({I}_1) + \varPsi _{f}({I}_4, {I}_6)= c({I}_1-3) + \sum \limits _{n=4,6} \psi ({I}_n), \end{aligned}$$

where *c* is the shear modulus of the ground matrix material, $\psi (\cdot )$ is the contribution to the strain-energy density from the fibres,

2 $$\begin{aligned} \psi \left( I_n\right) |_{(n=4,6)} = {\left\{ \begin{array}{ll} \frac{k_1}{2k_2}\left( \exp \left[ k_2(I_n-1)^2 \right] -1 \right) &{} I_n>1,\\ 0 &{} I_n \leqslant 1 , \end{array}\right. } \end{aligned}$$

and $k_1$ and $k_2$ are material parameters. For two fibre families aligned along the referential unit vector directions, $\mathsf {A}_{1}$ and $\mathsf {A}_{2}$, respectively, the invariants $I_n$ ($n=1,4,6$) can be written as

3 $$\begin{aligned} {I}_1 = {\text {tr}}\mathbf {{C}}, \qquad {I}_n = {\mathbf {C}} :\mathbf {M}_n,\quad n=4,6, \end{aligned}$$

where $\mathbf {C}=\mathbf {FF}^\mathrm{T}$ is the right Cauchy–Green tensor, $\mathbf {F}$ is the deformation gradient, $\mathbf {M}_4=\mathsf {A}_{1} \otimes \mathsf {A}_{1}$ and $\mathbf {M}_6=\mathsf {A}_{2} \otimes \mathsf {A}_{2}$ are the structure tensors derived from the fibre orientations. The invariants $I_4$ and $I_6$ are the square of the stretch ratios in the directions of $\mathsf {A}_{1}$ and $\mathsf {A}_{2}$, respectively. Therefore, $I_4$ and $I_6$ measure the deformation of the fibres.

### Damage

We use a linear cohesive traction-separation law to govern the behaviour of a torn part of the material [13]. There are two independent material parameters in the cohesive law, the maximum traction $T_\mathrm{{c}}$ that the material can bear without any damage, and the maximum separation or displacement $\Delta u_\mathrm{{c}}$ of surfaces while still maintaining cohesive forces between them.

Damage is activated at any point where the maximum principal stress $\sigma _1$ exceeds $T_\mathrm{{c}}$, i.e.

4 $$\begin{aligned} \sigma _1 > T_\mathrm{{c}} . \end{aligned}$$

Note that a compressive stress does not initiate damage.

The damage is activated at a material point when the damage criterion (4) is first satisfied. The cohesive traction-separation law is then used to relate surface tractions to jumps in displacement. Analogously to the stress–strain relation for elasticity, the cohesive law relates the traction *T* and separation $\Delta u$. There are no definitive experimental data on the traction-separation law for large arteries subject to dissection, and so, following Ferrara and Pandolfi [4], we have chosen a simple linear traction-separation law,

5 $$\begin{aligned} T = {\left\{ \begin{array}{ll} T_\mathrm{{c}} \left( 1- \Delta u/\Delta u_\mathrm{{c}} \right) &{}\qquad 0 \leqslant \Delta u \leqslant \Delta u_\mathrm{{c}}, \\ 0 &{} \qquad \Delta u > \Delta u_\mathrm{{c}}. \end{array}\right. } \end{aligned}$$

Generally, the fracture energy,

6 $$\begin{aligned} G_\mathrm{{c}} = T_\mathrm{{c}} \, \Delta u_\mathrm{{c}}/2, \end{aligned}$$

i.e. the total energy per unit area required to separate two surfaces by the critical separation $\Delta u_\mathrm{{c}}$, is specified in the computational model instead of $\Delta u_\mathrm{{c}}$.

### Extended finite element method

The initiation and propagation of a tear is implemented using the extended finite element method (XFEM) [3, 17]. Compared to a conventional FEM, the displacement in XFEM captures discontinuities, such as tears, by enriching the displacement field. Consider $\mathbf {X}$, a point in a FE model. Assume there is a discontinuity in the arbitrary domain discretised into *n*-node finite elements. In the XFEM, the approximation for calculation of the displacement for the point $\mathbf {X}$ within the domain is

7 $$\begin{aligned} \mathbf {u}^h(\mathbf {X}) = \mathbf {u}^\mathrm{FE}+\mathbf {u}^\mathrm{Enr} = \sum _{I=1}^n N_I(\mathbf {X}) \mathbf {u}_I + \sum _{J=1}^m N_J(\mathbf {X}) H(\mathbf {X}) \mathbf {a}_J, \end{aligned}$$

where $\mathbf {u}_I$ is the vector of regular degree of nodal freedom in the FEM, $\mathbf {a}_J$ is the vector of the additional *m* degrees of freedom and $H(\mathbf {X})$ is the discontinuous enrichment function defined for the set of nodes that the discontinuity has in its domain of influence. For example, to account for a displacement jump across a tear $\varGamma$, $H(\mathbf {X})$ may be defined as

8 $$\begin{aligned} H(\mathbf {X}) = {\left\{ \begin{array}{ll} 1 &{}\qquad \text {if } (\mathbf {X}-\mathbf {X}_\varGamma ) \cdot \mathbf {n} \geqslant 0, \\ -1 &{}\qquad \text {otherwise}, \end{array}\right. } \end{aligned}$$

where $\mathbf {X}_\varGamma \in \varGamma$ is the closest point to $\mathbf {X}$ on the tear and $\mathbf {n}$ is a outward-pointing normal vector of $\varGamma$ at $\mathbf {X}_\varGamma$. The first term on the RHS of (7) is the conventional finite element approximation, including all nodes, while the second term is the enrichment approximation which takes into account the existence of any discontinuities, including only nodes of elements bisected by the tear.

The main advantage of the XFEM is that the discontinuity at the tear is not explicitly expressed in the finite element mesh, but is placed in the interpolation of the solvable function $\mathbf {u}^h$. Therefore the mesh generation in the XFEM is independent of the tear. This also means that tear surfaces do not need to coincide with the interfaces between elements. The path of propagation of the tear is calculated based on the solution for the displacement field, the criterion (4) and the cohesive traction-separation law (5). The direction of propagation is determined to be normal to the direction of the first principal stress $\sigma _1$. We remark that although XFEM is a well-tested technique and well suited to our single tear problem, recent approaches such as eigen-erosion [20], and phase field [21] have also been developed, and could be more powerful in modelling branching and other complex fractures.

## Peeling-driven propagation

### Peeling-driven propagation in strips

#### Geometry and boundary conditions

**Fig. 1:**
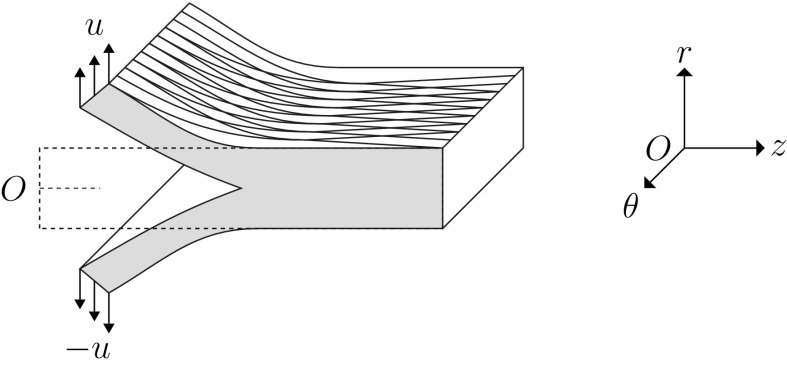
The sketch of the strip used in the peeling test [5], the cross-section (pink-shaded area) of which is the geometry used here. Displacement boundary conditions with same magnitude *u* but opposite directions are applied on the two arms

The peeling test performed in [5] is a representative experimental study to determine the failure properties of an arterial wall. In this test, a strip is cut from the arterial wall and the medial layer is isolated by removing the adventitia and intima. A tear is introduced at one end of the centre plane which is subject to displacement boundary conditions (Fig. 1). We solve this as a two-dimensional plane-strain problem.

The two families of fibres are distributed in the $\theta z$-plane and at $5^\circ$ to the *z*-axis [4]. Other parameters used in the constitutive laws are shown in Table 1. The strip has a width of 4 mm and height of 1.2 mm, the same geometry as used in [3–5]. The height represents the radial thickness of arterial wall, and the length of initial tear is assumed to be 0.4 mm.

**Table 1:** The material parameters for the samples used in the peeling test

*c* (kPa)	$k_1$ (kPa)	$k_2$	$T_\mathrm{{c}}$ (kPa)	$G_\mathrm{{c}}$ (N/m)
1	1	10	5	0.001

#### Results

**Fig. 2:**
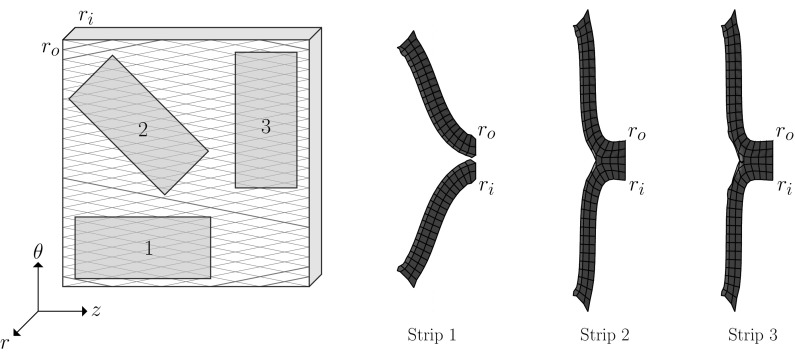
The failure status of three strips: red indicates the torn elements, blue undamaged elements, and green the cohesive zone. Strip 1 is completely torn when the displacement is $u=3.28$ mm and the other two strips can sustain loads until $u=4$ mm. The length of undamaged section in Strip 3 is greater than that in Strip 2

In order to find the preferred direction of the tear propagation, three strips with different orientations are simulated and the deformed configurations are shown in Fig. 2.

The simulations show that the circumferential strip (Strip 3) is most resistant to the tearing, since the length of the undamaged part is the longest. The orientation of collagen fibres is only $5^\circ$ to the *z*-axis direction (Fig. 2), therefore the stiffness is greatest along the *z*-axis. Figure 2 shows that the most favourable direction of propagation is along the *z*-axis. This agrees with the clinical observations of arterial dissection.

### Peeling-driven propagation of discs

#### Geometry and boundary conditions

**Fig. 3:**
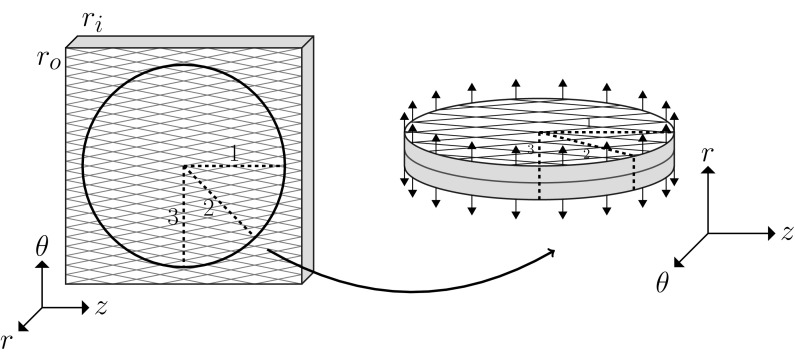
The radius of the disc is the same as the length of strips in Fig. 2. A circular tear (red line) is initialised at the edge of disc, of depth 0.1 times the disc radius, as in the simulations for strips. There is also a control case without fibres

We next simulate peeling of a disc. The disc is cut from the same sample of arterial wall as used for selection of strips (Fig. 3). The geometry of this disc is consistent with that of the strip. The radius is 4 mm and thickness is 1.2 mm. An initial tear with depth 0.4 mm along the radial direction is introduced. Thus, all three of the two-dimensional strips studied early are represented in this model, as shown by the cross-sections at 1, 2 and 3 in Fig.  3. Indeed, this three-dimensional model includes strips with all possible fibre orientations.

The boundary conditions are consistent with those in the simulation of peeling strips. The centres of top and bottom circles are fixed to avoid the rigid body motion. The displacements of $\pm \, 4$ mm in *r*-direction are applied, respectively, on the two edge surfaces (as shown by the pink areas in Fig. 3).

#### Results

**Fig. 4:**
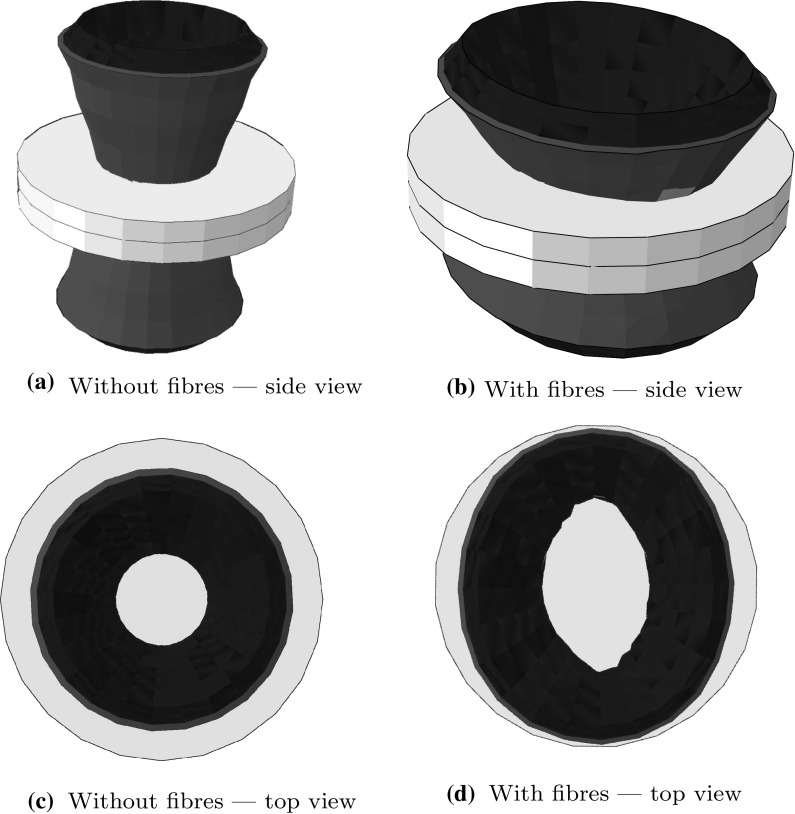
Peeling test on a disc. The grey regions are the undeformed configurations of the disc in Fig. 3, while the superimposed coloured ones show the deformed (peeled apart) configurations. The faces of the tear are red, and the deformed top and bottom surfaces are blue. In **c**, the undamaged central (grey) region of the fibre-free disc remains circular after peeling, while the undamaged central region of the fibrous disc **d** becomes elliptical with its major axis aligned along the mean fibre direction

The disc peeling tests are simulated both with and without fibres. As shown in the Fig. 4, the undamaged section of the fibre-free disc remains circular, while the undamaged part of the fibrous disc becomes elliptical. The short axis of the ellipse is in the *z*-direction, showing that the tear tends to propagate in the mean fibre direction. This is because (i) the the fibres resist deformation, so the strain and hence the stress is the greatest in the direction perpendicular to the mean fibre axis, and (ii) in the XFEM the tear propagates normal to the direction of the first principal stress (Sect. 2.3).

## Pressure-driven propagation

### Geometry and boundary conditions

The length and depth of the tear are both important factors in diagnosing the aortic dissection. In clinical practice, these data are generally extracted from the images, for example, from CT scans of the cross-section of the arterial wall. Here we study these effects on the propagation of arterial dissection through numerical simulations.

**Fig. 5:**
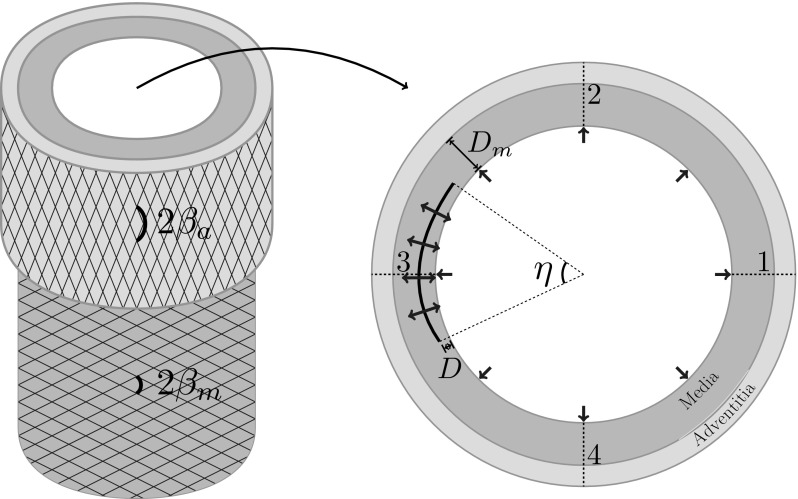
The cross-section of the two-layer model of the rabbit carotid arterial wall is considered as a plane-strain problem. The propagations of the initial tear (black arc) in the media subject to pressure (blue arrows) are simulated. The length of the tear is characterised by the central angel $\eta$. The depth of tear *D* is normalised by the thickness of the media $D_\mathrm{m}$, so the dimensionless depth is $d=D/D_\mathrm{m}$

Similar to clinical CT images, we consider the propagation of arterial dissection in a cross-section of arterial wall, as shown in Fig. 5. This is a two-dimensional plane-strain problem with the simplifying assumption that there is no variation in the axial direction. This example captures the planar propagation of the arterial dissection which can, however, *in vivo* assume a spiral path in the out-of-plane direction, e.g. from the aortic root to the aortic arch. We quantify the length of the initial tear using the central angle $\eta$. The initial tear is assumed to be located in the media and its depth from the inner surface is scaled on the thickness of the media, so that $d=0$ places the initial tear on the inner surface and $d=1$ means it is on the interface between media and adventitia. We shall simulate pressure-driven tear propagation for different values of $0^\circ<\eta <360^\circ$ and $0<d<1$.

Boundary conditions are applied to prevent rigid body motion: radii 1 and 3 (Fig. 5) are fixed in the *y*-direction so that only horizontal movement is allowed, while radii 2 and 4 are fixed in the *x*-direction so that only have the vertical movement is permitted.

We assume the pressure to be the same in both true and false lumina. As the pressure is gradually increased, we estimate the value of pressure when the dissection just starts to propagate at the tear tip, i.e. the critical pressure $p_\mathrm{{c}}$.

The two-layer arterial wall model uses parameters appropriate to a rabbit carotid artery [19]. Each layer is modelled using the HGO strain-energy function (1) but with different material parameters, as shown in Table 2. The geometric parameters are inner radius $R_\mathrm{i}$ = 0.74 mm, thickness of media $D_\mathrm{m}$ = 0.26 mm, and thickness of adventitia $D_\mathrm{a}$ = 0.12 mm.

**Table 2:** The material parameters in the HGO stressrain-energy function for the two-layer model of a rabbit carotid artery [19]

	*c* (kPa)	$k_1$ (kPa)	$k_2$	$\beta$ ($^\circ$)	$T_\mathrm{{c}}$ (kPa)	$G_\mathrm{{c}}$ (N/m)
Media	1.5	2.3632	0.8393	29	3.0	0.001
Adventitia	0.15	0.5620	0.7112	62	0.3	0.0001

### Simulations and results

#### Values of the tear depth *d* and length $\eta$

For convenience in the XFEM simulation, the values of *d* and $\eta$ chosen for simulations are such that the initial tear is always located within an element, and not along the element boundary. In practice, the best place to specify an initial tear is the bisector of an element. We choose the value of *d* to be $d_k=(k+0.5)\Delta d$, $k=0,\ldots ,n$, and $\Delta d=1.0/(n+1)$. From a set of simulations with $n=10$, we can estimate the critical pressure $p_\mathrm{{c}}$ for each value of *d*. In the following, the critical pressure $p_\mathrm{{c}}$ is non-dimensionlised by *c*, the shear modulus of the media.

#### Effects of *d* and $\eta$ on the critical pressure $p_\mathrm{{c}}$

**Fig. 6:**
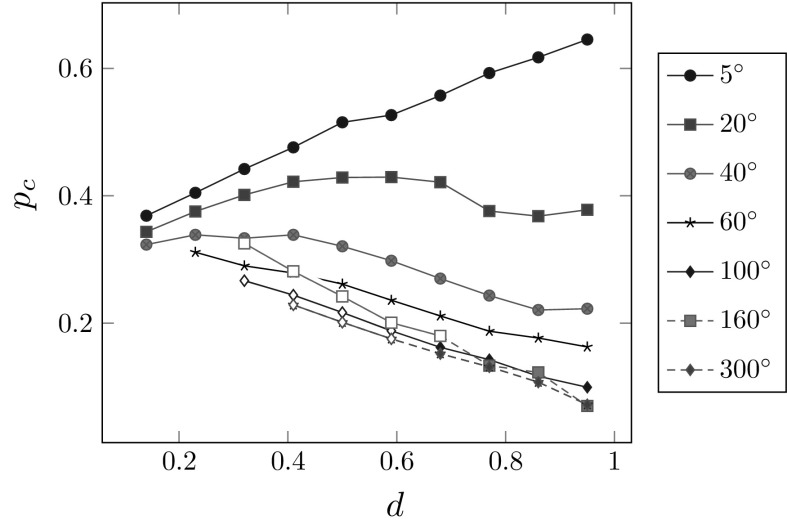
The dimensionless critical pressure $p_\mathrm{{c}}$ is plotted against the radial depth *d* for different values of $\eta$. Note for $\eta \ge 100^\circ$, the inner wall has a tendency to buckle, and the hollow versions of the symbols in the key indicate the cases in which the buckling occurs

**Fig. 7:**
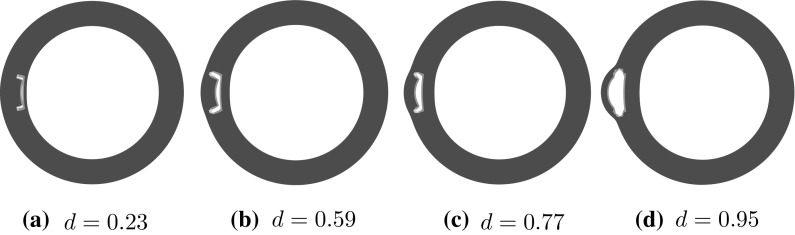
The representative shapes of deformed arterial wall with a short tear of $\eta =20^\circ$ for different radial depths *d*, subject to the critical pressure. The direction of initial propagation changes gradually from the radial (**a**) to the circumferential (**d**) as *d* increases

The variation of the critical pressure $p_\mathrm{{c}}$ against the radial depth *d* are plotted in Fig. 6, for different values of $\eta$. It is interesting to see that when the tear is very short, $\eta =5^\circ$, $p_\mathrm{{c}}$ increases monotonically with *d*. When the dissection is very long, i.e. $\eta \ge 60^\circ$, $p_\mathrm{{c}}$ decreases with *d* monotonically. However, when $\eta$ is between $20^\circ$ to $40^\circ$, the change is no longer monotonic. This may be related to the change in direction of tear propagation, as shown in Fig. 7.

Note that for a longer tear, e.g. $\eta \ge 100^\circ$, buckling of the inner wall (the material section between the lumen and tear) occurs, as shown by the hollow symbols in Fig. 6. This buckling phenomenon has also been observed in [13] for a tear located halfway through the media. Here, we find that buckling of the inner wall only occurs for tears with depth $d < 0.7$, and deeper tears are less likely to induce wall buckling.

Figure 7 shows the failure patterns of the deformed arterial wall with a tear $\eta =20^\circ$ subject to the critical pressure for different values of *d*. When $d=0.23$ in Fig. 7a, the tear propagates radially, but when $d=0.95$ in Fig. 7d it propagates circumferentially. When $d=0.59$ and 0.77, the direction of propagation is between the radial and the circumferential directions. Hence, the tear propagation undergoes a direction change, from the radial to the circumferential direction, as *d* increases. Similar trends are observed in simulations for $\eta =10^\circ$, $15^\circ$ and $25^\circ$.

Our results for $\eta >60^\circ$ show that $p_\mathrm{{c}}$ decreases almost linearly with the tear depth *d*. This agrees with the experimental observation by Tam et al. [9]. In that study, initial tears of different radial depths were introduced inside of a porcine aortic wall, which was then inflated under an increasing pressure. At the instantaneous time of tear propagation, the pressure was regarded as the critical pressure. In [9], this critical pressure is called the “propagation pressure”. The relationship showing that the critical pressure is a linearly decreasing function of the depth as shown in Fig. 2 of [9].

#### Effects of *d* and $\eta$ on the deformed shapes

**Fig. 8:**
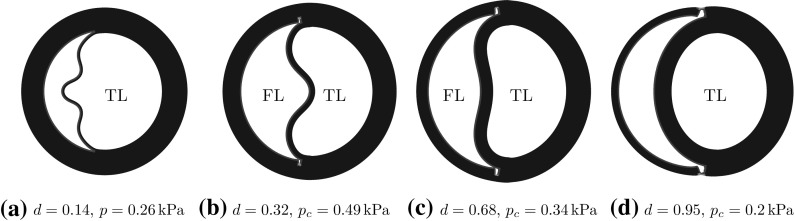
The representative shapes of deformed arterial wall with an initial tear $\eta =160^\circ$ for different radial depths *d*. The areas of the true lumen (TL) and the false lumen (FL) are marked. The buckling of the inner wall, the material between TL and FL, occurs, as shown in (**a**) and (**b**), which are similar to the CT scans of aortic dissection in patients in Fig. 9a and b, respectively

**Fig. 9:**
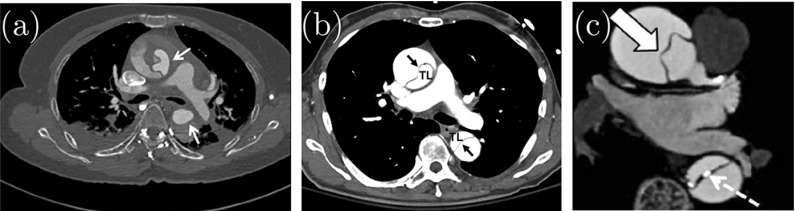
CT scans with buckling of the inner wall in type A aortic dissection: **a** Fig. 9 in [22], **b** Fig. 2 in [10], and **c** Fig. 2 in [23]

For a long tear, say, $\eta =160^\circ$, the inner wall starts to buckle when *d* is small. This is shown in Fig. 8a–c. Similar buckling patterns of the inner wall have been observed in CT scans of acute aortic dissections. For example, the buckling mode of the inner wall in Fig. 8a is similar to that observed in the CT scan in Fig. 9a, and the buckling mode in Fig. 8b is similar to the CT scan of the ascending aorta in Fig. 9b. Although our model is simplified, it is encouraging that the model can reproduce qualitatively similar patterns observed in clinics.

Comparison of the deformed shapes of the arterial wall with different tear depth *d* shows that the radial depth of a tear plays an important role in wall deformation, which in turn determines the likelihood of tear propagation. The results in Fig. 8a–d show that the buckling mode is the highest in (a), and this tear does not propagate. The buckling mode decreases as *d* increases from (a) to (c), with no buckling in (d), when the tear is deepest.

**Fig. 10:**
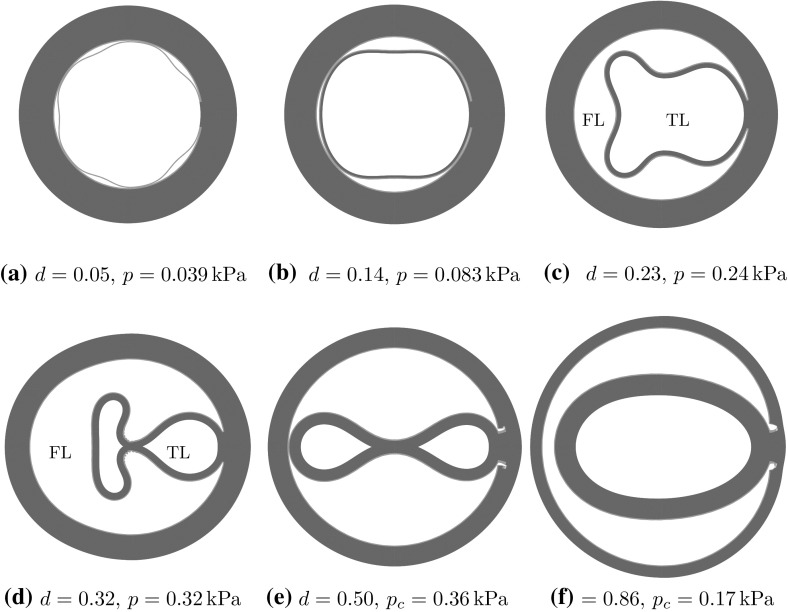
The representative deformed shapes of arterial wall with a long tear $\eta =340^\circ$ for different radial depth *d*. When the depth is small (**a**–**d**), the propagation does not occur. Instead, buckling of the inner wall occurs, and the true lumen is collapsed in (**d**) and (**e**). The shape of the buckled inner wall in (**c**) is similar to that in the CT scan of the aortic dissection for a patient in Fig. 9c

In addition, we plot the deformed shapes of the arterial wall with a longer tear $\eta =340^\circ$ for different values of *d* in Fig. 10. In these results, we also observe that the buckling mode of the inner wall with $d=0.23$ in Fig. 10c is similar to that in the CT scan of the aortic dissection in Fig. 9c [23]. For a tear with $d=0.32$ and 0.50 (Fig. 10d, e), the *collapse of the true lumen* occurs as a result of buckling of the inner wall.

## Conclusions

We have developed computational models to understand arterial dissection. In this paper, the stable displacement-peeling-driven and unstable pressure-driven tear propagation are simulated. The simulations for the peeling test show the tear prefers to propagate along the direction of material axial with the maximum stiffness, which is determined by the fibre orientation.

We also investigate the effects of depth and length on the critical condition and tear propagation under pressure for a two-layer plane-strain arterial wall model. We found that the critical propagation pressure is greater for deeper and shorter tears. Our model can also reproduce a range of inner wall buckling patterns akin to the clinical observations using different tear lengths and depths, despite the simplifying assumption of plane-strain that neglects variation and propagation in the axial direction. Our results suggest that a long and shallow tear is more likely to induce the inner wall buckling. We hypothesise that a buckled state redistributes the energy and stresses within the wall, and this may work to slow down the tear propagation. This is supported by the observation that a deep tear without buckling tends to propagate with a lower critical pressure.
